# Negative controls: Concepts and caveats

**DOI:** 10.1177/09622802231181230

**Published:** 2023-06-20

**Authors:** Bas BL Penning de Vries, Rolf HH Groenwold

**Affiliations:** 1 Julius Center for Health Sciences and Primary Care, Utrecht University Medical Center, Utrecht, The Netherlands; 2 Department of Clinical Epidemiology, 4501 Leiden University Medical Center, Leiden, The Netherlands; 3 Department of Biomedical Data Sciences, 4501 Leiden University Medical Center, Leiden, The Netherlands

**Keywords:** Causal inference, unmeasured confounding, negative control exposures, negative control outcomes

## Abstract

Unmeasured confounding is a well-known obstacle in causal inference. In recent years, negative controls have received increasing attention as a important tool to address concerns about the problem. The literature on the topic has expanded rapidly and several authors have advocated the more routine use of negative controls in epidemiological practice. In this article, we review concepts and methodologies based on negative controls for detection and correction of unmeasured confounding bias. We argue that negative controls may lack both specificity and sensitivity to detect unmeasured confounding and that proving the null hypothesis of a null negative control association is impossible. We focus our discussion on the control outcome calibration approach, the difference-in-difference approach, and the double-negative control approach as methods for confounding correction. For each of these methods, we highlight their assumptions and illustrate the potential impact of violations thereof. Given the potentially large impact of assumption violations, it may sometimes be desirable to replace strong conditions for exact identification with weaker, easily verifiable conditions, even when these imply at most partial identification of unmeasured confounding. Future research in this area may broaden the applicability of negative controls and in turn make them better suited for routine use in epidemiological practice. At present, however, the applicability of negative controls should be carefully judged on a case-by-case basis.

## 1. Introduction

In epidemiological research on causal effects, there are often concerns that one or more assumptions – such as exchangeability, no measurement error, or assumptions about missing data – are violated. In efforts to lessen these concerns, it has long been suggested that auxiliary variables be used that have a known (e.g. null) causal relation with the exposure or outcome of interest.^1–3^ Observing an association that contradicts the belief in a causal null might alert the analyst to violations of the assumptions underlying the methods used in the study. Auxiliary variables known to be causally unrelated to the variables of primary interest are called negative controls and have the potential in bias detection as well as partial or complete bias correction in epidemiological research.^4^

Applications of negative controls in epidemiological research are diverse. Dusetzina et al.^5^ identified 11 studies that used a negative control exposure, negative control outcome, or both in studies on various topics, ranging from peri-operative beta-blocker use and the risk of acute myocardial infarction to proton-pump inhibitors and community-acquired pneumonia risk. Schuemie et al.^6^ studied as many as 37 and 67 negative control exposures in two example studies on isoniazid use and acute liver injury and on selective serotonin reuptake inhibitor use and gastrointestinal bleeding, respectively. Increased attention for negative controls is exemplified by mention in, for example, the RECORD-PE reporting guideline for pharmacoepidemiological studies and the STROBE-MR guideline for Mendelian randomisation studies.^7,8^

In recent years, negative controls have received increasing attention in the epidemiological and statistical literature. The literature on how to leverage negative controls in studies on causal effects has rapidly expanded and several authors have argued that negative controls should be more commonly employed.^2,9,4^ This article aims to complement these efforts to increase the more routine implementation of negative controls with a discussion about a selection of caveats. Although we zoom in on the limitations of negative control methods, it should be noted that other methods (e.g. instrumental variable methods and conventional adjustment for a minimally sufficient set of covariates) are similarly subject to limitations and need not be universally preferred over negative controls. Focusing on the use of negative controls to address possible violations of the exchangeability assumption, that is, the assumption of no unmeasured confounding, we begin with a brief review of relevant definitions and discuss assumptions for bias detection. We then review methods for bias correction and study their sensitivity to assumption violations.

## 2. Negative controls

**Figure 1.**
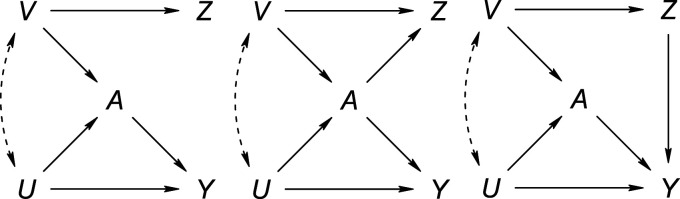
Causal directed acyclic graphs of settings where Z is a negative control outcome (left), a negative control exposure (middle) or both (right). The absence of an arrow denotes the absence of a direct causal link. However, the presence of an arrow need not represent the presence of a direct causal link. Dashed double-headed arrows represent the marginal dependence of the (sets of) variable(s) that they connect, for example, through a common cause.

A negative control outcome (NCO) is a variable that is not causally affected by the exposure of interest A .^10,4^ Likewise, a negative control exposure (NCE) is a variable that does not causally affect the outcome of interest Y , except possibly through the exposure of interest.^4^ The causal directed acyclic graphs of Figure 1 (discussed later in this section) give examples of settings where a variable Z classifies as an NCO, an NCE or both. Given the absence of a direct causal effect of exposure A on an NCO Z or of NCE Z on outcome Y , any observed association between A and an Z , or between an Z and outcome Y given A , must be spurious. Leveraging negative controls involves translating information about such spurious associations into information about the spuriousness of associations between the primary exposure and outcome variables of interest.

### 2.1. Negative controls for unmeasured confounding detection

Let Y(a) denote the outcome that would be realized had exposure A been set to a . Together with causal consistency (i.e. Y(a)=Y if A=a ) and positivity, epidemiologists often seek to invoke the exchangeability (or unmeasured confounding) condition Y(a)⊥⊥A (possibly within levels of a collection of observed variables) to establish identifiability of the effect of exposure A on outcome Y .^11^ In observational studies, however, it is seldom evident that the exchangeability condition, E, for the exposure-outcome relation of interest is achieved. A key idea of negative controls is to find a ‘control’ statement, C, that translates into information about E and which is more easily verified or refuted.

Control statement C may refer to the absence of bias of a measure of the association between A and Y and the NCO or NCE variable, respectively. Knowing that any control association is noncausal renders the control statement empirically verifiable. If C implies E, then a null finding for the control statement would imply conditional exchangeability for the exposure–outcome relation of interest. Conversely, if E implies C, evidence of the bias of the control association corroborates the existence of unmeasured confounding.

### 2.2. Caveats in the use of negative controls to detect unmeasured confounding

There are a number of caveats concerning the use of negative controls for confounding detection. These caveats mainly concern the link between the control statement and exchangeability for the exposure–outcome relation of interest. Unfortunately, the extent to which one confers information about the other need not be evident.^12^ A biased negative-control association need not imply unmeasured confounding for the exposure–outcome relation of interest and neither is the converse true generally.

First, while most applications of negative controls assume that confounding is the only source of bias, in reality, it may be one of potentially many sources of bias. A spurious negative control association could have resulted, at least in part, from collider stratification, measurement error, or violations of assumptions about missing data.^9^ Even if unmeasured confounding for the negative control association implies unmeasured confounding for the exposure–outcome relation of interest, a biased negative control association need not be a reflection of unmeasured confounding. Conversely, a (near) null finding could be the result of opposing biases, masking the presence of unmeasured confounding. In other words, negative controls are a tool that may lack both specificity and sensitivity with respect to the type(s) of bias they are to detect.

Lipsitch et al.^2^ suggested a principle for establishing a link that is based on the extent to which common causes of A and Y overlap with the common causes of the exposure or outcome and the negative control variable. Clearly, for an NCO, with complete overlap (e.g. V=U in Figure 1), the set of common causes of A and Y is empty if and only if the set of common causes of A and the NCO is empty. However, null values for certain measures of the effect of A on an NCO or of an NCE on Y need not imply that the set of unobserved common causes is empty, or, therefore, that there is conditional exchangeability for the primary exposure–outcome relation. Indeed, near null values may be the result of partially opposing confounding effects (or, more generally, opposing biases), and the relative effects may be different for the NCO versus the primary outcome Y .

With finite samples rather than complete knowledge of the theoretical or population distribution, sampling variability becomes relevant too, making it more important to acknowledge the distinction between absence of evidence and evidence of absence.^13^ With finite samples, proving the null hypothesis of a null negative control association is impossible. Even if ‘highly’ powered studies cannot detect bias for the negative control relation, it may be injudicious to assume that the available data are sufficient to adequately control for confounding of the primary relation of interest, because a small degree of bias for the former relation may be associated with a substantial degree of bias for the latter. Sample size and power considerations are often ignored or left at secondary importance. While some papers have considered the power of negative control tests,^1,14^ it is typically ignored how the negative control association relates to the extent of bias for the exposure–outcome relation of interest, yet high power to detect ‘small departures’ from exposure-NCO or NCE-outcome independence need not imply high power to detect small bias due to unmeasured confounding of the primary relation of interest. What are considered ‘small departures’ should therefore depend on the relationship between bias of the negative control association and the bias of the exposure–outcome relation of interest, or, likewise, depending on the link between the control statement C and the exchangeability condition E (as outlined in Section 2.1). Conversely, even if there is evidence of the contrary to the negative control null hypothesis, the bias due to uncontrolled confounding for the primary exposure–outcome relation may not be meaningful. In any case, it is important to consider the relative size of the biases in the negative control and primary exposure–outcome relations.

## 3. Negative control methods for uncontrolled confounding adjustment

The more recent literature on negative controls has considered how and under what conditions negative controls can be leveraged to partially or fully identify target causal quantities rather than merely the presence of bias. Lipsitch et al.^2^ give conditions for valid inference about the direction of bias and thus for partial identification of the target causal quantity. These conditions are reviewed in Supplemental Appendix A. In what follows, we review three methods for full identification: the control outcome calibration approach (COCA), the (generalized) difference-in-difference (DiD) approach, and the double-negative control approach. Proofs of identification are given in Supplemental Appendix B for completeness. For each of the methods, we illustrate the potential impact of assumption violations on the identifiability of the targeted quantity. Throughout, departures from identification are termed bias.

### 3.1. Control outcome calibration approach

#### 3.1.1. Identification

It may be tempting to regard the confounded association between the exposure of interest and an NCO as a direct measure of bias for the exposure–outcome effect of interest. However, it cannot generally be assumed that the direction or magnitude of bias is the same for the two relations. As an alternative to the restrictive and probably unrealistic ‘bias equivalence’ assumption, that is, the assumption of equality between the confounded negative control association and the bias due to unmeasured confounding of the exposure–outcome effect of interest, Tchetgen Tchetgen^10^ proposed the COCA. The assumption of ‘bias equivalence’ would especially likely be violated if the NCO and primary outcome are measured on different scales and the bias is bounded differently depending on the scale, such as would be the case if the NCO was binary and the primary outcome continuous. The COCA leverages an NCO to adjust for unmeasured confounding without requiring that the NCO and primary outcome Y are measured on similar scales.

The next result, due to Tchetgen Tchetgen,^10^ describes a regression-based approach to implementing the COCA, which – characteristically of the COCA – relies on the assumption that a (set of) counterfactual primary outcome(s) of interest is sufficient to render the NCO conditionally independent of the exposure of interest. Some intuition behind this approach may be obtained upon noting that the counterfactual outcomes may well capture information about baseline covariates and therefore serve as a proxy for unobserved pre-exposure variables that are predictive of the NCO. The reasoning rests on the assumption that the same covariates that explain the lack of exchangeability for the outcome of interest also explain the confounding of the exposure–NCO relation. However, even then it is not evident nor guaranteed that the counterfactual outcome proxy is sufficient to render the NCO and exposure conditionally independent.

Theorem 1 **A regression-based approach to implementing the COCA under rank preservation**

Suppose that the following conditions hold for all levels a of A :

∙ *Consistency:* Y(a)=Y if a=A .

∙ *Rank preservation:* for some constant θ , Y(0)=Y(a)−θa .

∙ *Exposure-NCO independence given counterfactual outcome:* Z⊥⊥A|Y(0) .

∙ *NCO model:* for known one-to-one model link g , $g(E[Z|A,Y])=\beta_{0}+\beta_{1}A+\beta_{2}Y$ , where $\beta_{0},\beta_{1},\beta_{2}$ are identified by a regression of Z on A and Y , and $\beta_{2}\neq 0$ .

Then, E[Y(a)−Y(a−1)]=θ is identified by $-\beta_{1}/\beta_{2}$ .

Because counterfactual outcome Y(0) may not fully account for the unmeasured confounding between the exposure and NCO, it is important that the impact of assumption violations be gauged. To this end, Tchetgen Tchetgen described a sensitivity analysis,^10^ given below in Theorem 2, for the special case of Theorem 1, where g is the identity link and A is a linear combination of Y(0) and an error term Δ . When the sensitivity parameter ( ρ ) is set to 0 , it is implicitly assumed that the NCO and exposure of interest are independent given counterfactual outcome Y(0) (because χ is independent of (A,Y) and therefore of Y(0) ) and, so, the result of Theorem 1 is recovered.

Theorem 2 **Sensitivity analysis for violations of Z⊥⊥A|Y(0)
**

Suppose the following conditions hold for all levels a of A :

∙ *Consistency:* Y(a)=Y if a=A .

∙ *Rank preservation:* for some constant θ , Y(0)=Y(a)−θa .

∙ *Conditional exposure-NCO independence:* Z⊥⊥A|(Y(0),Δ) .

∙ *Exposure model:* $A=\alpha_{0}+\alpha_{1}Y(0)+\Delta$ .

∙ *NCO model:* $Z=\beta_{0}+\beta_{1}Y(0)+\rho \Delta +\chi$ , χ⊥⊥(A,Y) .

Then, $E[Z|A,Y]=\beta_{0}^{*}+\beta_{1}^{*}A+\beta_{2}^{*}Y$ for some $\beta_{0}^{*},\beta_{1}^{*},\beta_{2}^{*}$ , and if parameters $\beta_{1}^{*},\beta_{2}^{*}$ are identified (by a regression of Z on A and Y ) and $\beta_{2}^{*}\neq 0$ , then $\theta =(\beta_{1}^{*}-\rho )/\beta_{2}^{*}$ .

Through the rank preservation assumption, Theorem 1 relies also on the strong assumption that all counterfactual outcomes of an individual are deterministically linked. A prerequisite of this assumption is that the within-person ranks of counterfactuals are the same for all individuals. In the next section, we consider violations of this assumption. However, as Theorem 3 states, in the special case where the outcome and exposure of interest are binary, there should be no concern about violations of this assumption as it can be dropped entirely.^10^

Theorem 3 **COCA for binary primary outcome and exposure**

Suppose that the following conditions hold:

∙ *Consistency:* Y(a)=Y if a=A

∙ *Positivity:* 0<Pr(A=a,Y=y) for y=0,1 .

∙ *Exposure-NCO independence given counterfactual outcome:* Z⊥⊥A|Y(a) .

∙ *Non-zero denominator:* E[Z|A=a,Y=1]−E[Z|A=a,Y=0]≠0 .

Then,

$$\begin{array}{rl} E[Y(a)] & =E[Y|A=a]\mathrm{Pr}(A=a)+\frac{E[Z|A=1-a]-E[Z|A=a,Y=0]}{E[Z|A=a,Y=1]-E[Z|A=a,Y=0]}\mathrm{Pr}(A=1-a) \end{array}$$

If the assumptions of Theorem 3 are met for a=1 , the average treatment effect among the treated (ATT) E[Y−Y(0)|A=1] is identified. For identification of the average treatment effect (ATE) E[Y(1)−Y(0)] , the result requires that the assumptions are met for a=0,1 . We will consider violations of these assumptions in the next section.

#### 3.1.2. Sensitivity to assumption violations

In this subsection, we consider the sensitivity of the COCA to assumption violations. In particular, we illustrate the potential impact of deviating from rank preservation and of violating the assumption that counterfactual outcome Y(0) renders the exposure and NCO conditionally independent. While the classical measurement error in the outcome does not hamper inference in terms of bias in the classical linear regression setting, we also illustrate that this form of measurement error does result in bias of the COCA.

First, to illustrate the potential impact of deviating from rank preservation, consider the setting where A is binary and where the following models hold:

(1) $$\begin{array}{r} \begin{array}{rl} \theta |A & ∼\mathrm{Normal}(E[\theta ],\sigma_{\theta }^{2}),\\ Y(0)|A,\theta & ∼\mathrm{Normal}(\alpha_{0}+\alpha_{1}A,\sigma_{Y}^{2}),\\ Y=Y(A) & =Y(0)+\theta A,\\ Z|(A,\theta ,Y(0)) & ∼\mathrm{Normal}(\gamma_{0}+\gamma_{1}Y(0),\sigma_{Z}^{2}). \end{array}\} \end{array}$$

A standard implementation of the COCA as per Theorem 1 yields $\hat{\theta }=-{\hat{\beta }}_{1}/{\hat{\beta }}_{2}$ , where ${\hat{\beta }}_{1}$ and ${\hat{\beta }}_{2}$ are the coefficients for A and Y of an ordinary least squares regression of Z on A and Y .

Given a value of the ATE (i.e. E[θ] ), the parameter values are fully determined under models (1) by the joint distribution of the observed variables A,Y,Z (Supplemental Appendix C). In particular, given a fixed distribution of (A,Y,Z) , the variance of the individual effects Y(1)−Y(0) (i.e. $\mathrm{Var}(\theta )=\sigma_{\theta }^{2}$ ) and the ATE are linearly related via

$$\begin{array}{rl} \mathrm{Var}(\theta ) & =\frac{\mathrm{Var}(A)\mathrm{Var}(Y)-\mathrm{Cov}(A,Y{)}^{2}}{\left(\mathrm{Var}(A)+E[A{]}^{2})\mathrm{Cov}(A,Z\right)}({\hat{\beta }}_{1}-{\hat{\beta }}_{2}E[\theta ]) \end{array}$$

(Supplemental Appendix C). For values of the ATE between − 4 and 2, we chose parameter values such that the distribution of (A,Y,Z) has marginal means E[A]=0.25 , E[Y]=0 and E[Z]=0 , and covariance matrix

(2) $$\begin{array}{r} \left[\begin{array}{ccc} 3/16 & 1/2 & 1/2\\ 1/2 & 3 & 2\\ 1/2 & 2 & 4 \end{array}\right] \end{array}$$

**Figure 2.**
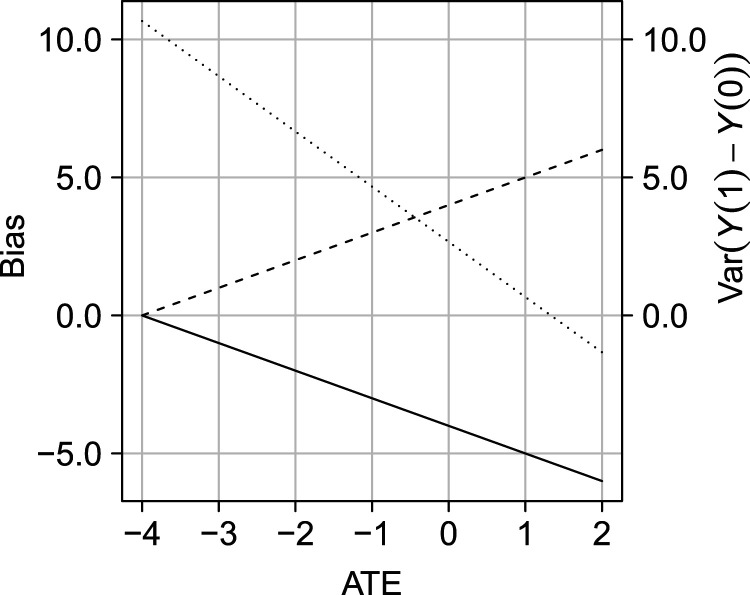
Illustration of the effect of violating the rank preservation assumption on the difference between the quantity identified by the COCA and the ATE (bias of COCA; solid line) and the difference between E[Y|A=1]−E[Y|A=0] and the ATE (bias of crude analysis; dotted line). The dashed line depicts the relation between the variance of individual exposure–outcome effects Y(1)−Y(0) and the mean E[Y(1)−Y(0)] (the ATE) under a fixed observed data distribution; the solid line describes the relation between the ATE and the bias of the implementation of the COCA. COCA: control outcome calibration approach; ATE: average treatment effect.

Figure 2 shows the bias of the COCA for the ATE. As shown, the magnitude of the bias is zero under rank preservation but increases linearly with an increasing variance of individual exposure–outcome effects.

In illustrating the sensitivity of the COCA against violations of rank preservation, it was assumed that the other assumptions were maintained. We now turn to the assumption of Exposure–NCO independence given counterfactual outcome Y(0) and likewise assume that all other assumptions, including rank preservation, are met. In particular, we consider the setting where Y(0) is the sum of two independent variables $U_{1},U_{2}$ . By assuming the following models, we also stipulate that some (albeit not necessarily the same) linear combination $\alpha_{0}^{\prime }+\alpha_{1}^{\prime }U_{1}+\alpha_{2}^{\prime }U_{2}$ is sufficient to render the exposure of interest and NCO conditionally independent:

(3) $$\begin{array}{r} \begin{array}{rl} U_{1} & ⊥\;⊥U2,\\ A|(U_{1},U_{2}) & ∼\mathrm{Normal}(\alpha_{0}+\alpha_{1}U_{1}+\alpha_{2}U_{2},\sigma_{A}^{2}),\\ Y=Y(A) & =U_{1}+U_{2}+\theta A,\;\theta \text{ constant},\\ Z|(U_{1},U_{2},A,Y) & ∼\mathrm{Normal}(\alpha_{0}^{\prime }+\alpha_{1}^{\prime }U_{1}+\alpha_{2}^{\prime }U_{2},\sigma_{Z}^{2}) \end{array}\} \end{array}$$

**Figure 3.**
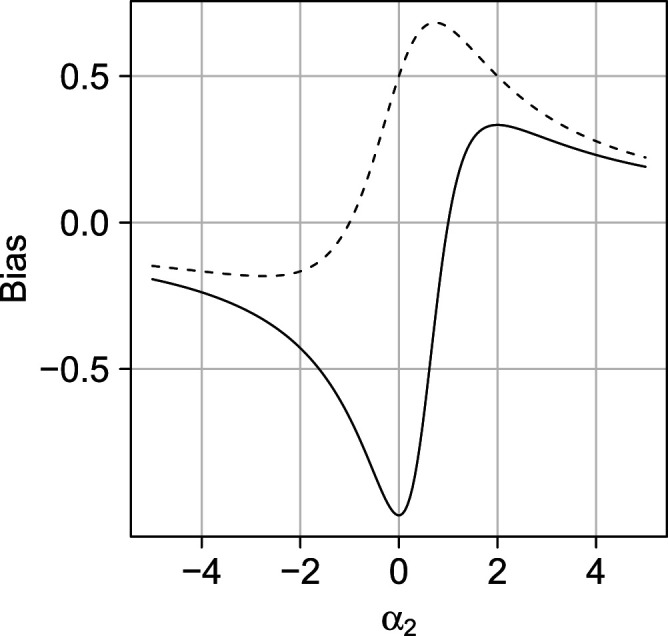
Illustration of the potential impact of violating the assumption that the NCO and exposure of interest are independent given counterfactual outcome Y(0) . The bias of the COCA (COCA − ATE) is given by the solid line; the bias of a crude analysis, Cov(Y,A)/Cov(A) , by the dashed line. NCO: negative control outcome; COCA: control outcome calibration approach; ATE: average treatment effect.

Variables $U_{1}$ and $U_{2}$ can be viewed as common causes of the NCO and the exposure and outcome of interest. Again, the COCA identifies the quantity $\hat{\theta }=-{\hat{\beta }}_{1}/{\hat{\beta }}_{2}$ based on an ordinary least squares regression of NCO Z on A and Y , but this quantity is not generally equal to θ . Figure 3 shows the asymptotic bias (departure from identification of the ATE) of the COCA plotted against $\alpha_{2}$ over the interval (−5,5) for the special case where $U_{1}$ and $U_{2}$ take the standard normal distribution and where $\alpha_{0},\alpha_{0}^{\prime },\alpha_{2}^{\prime }=0$ , $\alpha_{1},\sigma_{A}^{2},\sigma_{Z}^{2}=1$ and $\alpha_{1}^{\prime }=2$ . The bias is zero only when counterfactual outcome Y(0) is proportional to the linear combination of common causes $U_{1}$ and $U_{2}$ that renders the NCO and exposure of interest conditionally independent.

With $\alpha_{2},\alpha_{2}^{\prime }=0$ , models (3) imply the same joint distribution of observed variables A,Y,Z as models (4):

(4) $$\begin{array}{r} \begin{array}{rl} U_{1} & ⊥\;⊥U2,\\ A|(U_{1},U_{2}) & ∼\mathrm{Normal}(\alpha_{0}+\alpha_{1}U_{1},\sigma_{A}^{2}),\\ Y(A) & =U_{1}+\theta A,\;\theta \text{ constant},\\ Y & =Y(A)+U_{2},\\ Z|(U_{1},U_{2},A,Y) & ∼\mathrm{Normal}(\alpha_{0}^{\prime }+\alpha_{1}^{\prime }U_{1},\sigma_{Z}^{2}) \end{array}\} \end{array}$$

An important difference between (3) and (4) is that the consistency assumption is violated (provided that $\mathrm{Var}(U_{2})>0$ ). The observed outcome Y is now the sum of the outcome of interest Y(A) and an independent mean-zero error term. Figure 3 therefore also illustrates that the validity of the COCA also critically rests on the absence of classical measurement error in the outcome. At $\alpha_{2}=0$ , Figure 3 gives the bias of the COCA under (4) with the values for the parameters given above. Although ATE θ may not be identified in the presence of classical measurement error, in Supplemental Appendix C, partial identification bounds are derived for θ .

### 3.2. DiD approach

#### 3.2.1. Identification

The DiD approach proposed by Sofer et al.^15^ is an alternative approach to the COCA and does not assume rank preservation, nor does it require that the counterfactual outcome Y(0) renders the NCO and exposure of interest conditionally independent. Instead, the approach relies on bias equivalence for the primary exposure–outcome relation and the exposure–NCO relation. The simplest version of the DiD approach identifies the ATT under additive equi-confounding, as stated in Theorem 4, via the difference between the crude difference in primary outcome means and the bias of the exposure–NCO relation.

Theorem 4 **DiD approach for the ATT under additive equi-confounding**

Suppose that the following conditions hold for all levels a=0,1 :

∙ *Consistency:* Y(a)=Y if a=A .

∙ *Additive equi-confounding:* E[Y(0)|A=1]−E[Y(0)|A=0]=E[Z|A=1]−E[Z|A=0] .

Then, E[Y(1)−Y(0)|A=1]=(E[Y|A=1]−E[Y|A=0])−(E[Z|A=1]−E[Z|A=0]).

**Figure 4.**
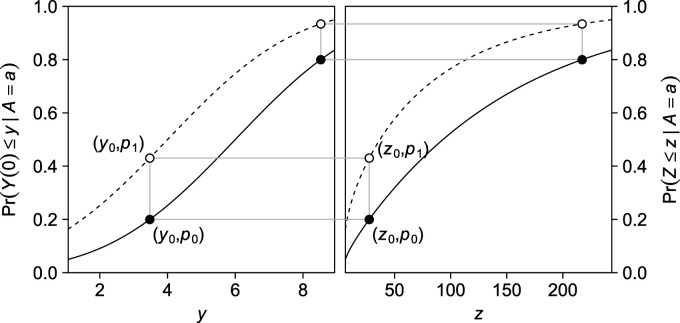
Example of quantile–quantile equi-confounding. Dashed curves represents a=1 , solid curves a=0 . There is quantile–quantile equi-confounding because for every two points $\left(y_{0},p_{0}\right)$ and $\left(y_{0},p_{1}\right)$ on the solid and dashed curves, respectively, of the left panel, there exists $z_{0}$ such that $\left(z_{0},p_{0}\right)$ and $\left(z_{0},p_{1}\right)$ lie on the solid and dashed curves, respectively, of the right panel; quantiles $y_{0}$ and $z_{0}$ need not be the same.

Additive equi-confounding is relatively easy to interpret. However, the assumption may be particularly likely to be violated when primary outcome Y and NCO Z are measured on different scales (e.g. one is a binary variable, the other continuous). A generalized DiD approach still identifies the ATT under a different constraint on the dependence between Y(0) and A in relation to the dependence between Z and A . In particular, Theorem 5, based on Sofer et al.,^15^ relies on quantile–quantile equi-confounding, an example of which is depicted in Figure 4.

Theorem 5 **Generalized DiD approach for the ATT under quantile–qualine equi-confounding**

Suppose that the following conditions hold for all levels a=0,1 :

∙ *Consistency:* Y(a)=Y if a=A .

∙ *Quantile-quantile equi-confounding:* $F_{0}(F_{1}^{-1}(p))=G_{0}(G_{1}^{-1}(p))$ for all p∈[0,1] , where $F_{a}(y)=\mathrm{Pr}(Y(0)\leq y|A=a)$ , $F_{a}^{-1}(p)=\min\{y:p\leq F_{a}(y)\}$ , $G_{a}(z)=\mathrm{Pr}(Z\leq z|A=a)$ , $G_{a}^{-1}(p)=\min\{z:p\leq G_{a}(z)\}$ .

∙ $F_{1}$ is strictly increasing.

Then, $E[Y(1)-Y(0)|A=1]=E[Y|A=1]-E[F_{0}^{-1}(G_{0}(G_{1}^{-1}(V)))]$ , where V∼Uniform[0,1] .

#### 3.2.2. Sensitivity to assumption violations

We now give a simple setting where neither additive nor quantile–quantile equi-confounding is guaranteed to hold. The setting is characterized by two common causes $U_{1},U_{2}$ of the primary exposure and outcome and of the NCO. As before, we allow the relative effects of these common causes to differ between exposure, primary outcome and NCO, and we suppose that the following models hold:

(5) $$\begin{array}{r} \begin{array}{rl} A & ∼\mathrm{Bernoulli}(p_{A}),\\ U_{1}|A & ∼\mathrm{Normal}(\alpha_{0}+\alpha_{1}A,\sigma_{1}^{2}),\\ U_{2}|(U_{1},A) & ∼\mathrm{Normal}(\alpha_{0}^{\prime }+\alpha_{1}^{\prime }A,\sigma_{2}^{2}),\\ Y(0)|(U_{1},U_{2},A) & ∼\mathrm{Normal}(U_{1}+U_{2},\sigma_{Y}^{2}),\\ Y=Y(A) & =Y(0)+\theta A,\;\theta \;\text{constant},\\ Z|(U_{1},U_{2},A,Y(0)) & ∼\mathrm{Normal}(\beta_{0}+\beta_{1}U_{1}+\beta_{2}U_{2},\sigma_{Z}^{2}). \end{array}\} \end{array}$$

Parameters $\alpha_{1},\alpha_{1}^{\prime },\beta_{1},\beta_{2}$ control the dependence (confounding), through $U_{1}$ and $U_{2}$ , between A and Y(0) and between A and NCO Z ; in the special case where these parameters take the value 0, there is no confounding. The models of (5) imply

$$\begin{array}{rl} Y(0)|A & ∼\mathrm{Normal}((\alpha_{0}+\alpha_{0}^{\prime })+(\alpha_{1}+\alpha_{1}^{\prime })A,\sigma_{1}^{2}+\sigma_{2}^{2}+\sigma_{Y}^{2})\\ Y|A & ∼\mathrm{Normal}((\alpha_{0}+\alpha_{0}^{\prime })+(\alpha_{1}+\alpha_{1}^{\prime }+\theta )A,\sigma_{1}^{2}+\sigma_{2}^{2}+\sigma_{Y}^{2})\\ Z|A & ∼\mathrm{Normal}((\beta_{0}+\beta_{1}\alpha_{0}+\beta_{2}\alpha_{0}^{\prime })+(\beta_{1}\alpha_{1}+\beta_{2}\alpha_{1}^{\prime })A,\beta_{1}^{2}\sigma_{1}^{2}+\beta_{2}^{2}\sigma_{2}^{2}+\sigma_{Z}^{2}) \end{array}$$

Implementing the DiD for the ATT θ would therefore identify, under (5), the quantity

$$\begin{array}{rl} \left(E[Y|A=1]-E[Y|A=0])-(E[Z|A=1]-E[Z|A=0]\right) & =(1-\beta_{1})\alpha_{1}+(1-\beta_{2})\alpha_{1}^{\prime }+\theta , \end{array}$$

with a bias of $(1-\beta_{1})\alpha_{1}+(1-\beta_{2})\alpha_{1}^{\prime }$ . The generalized DiD would instead identify

$$\begin{array}{rl} E[Y|A=1]-E[F_{0}^{-1}(G_{0}(G_{1}^{-1}(V)))] & =(\alpha_{0}+\alpha_{0}^{\prime })+(\alpha_{1}+\alpha_{1}^{\prime }+\theta )-\int_{-\infty }^{+\infty }F_{0}^{-1}(G_{0}(G_{1}^{-1}(p)))\;dp, \end{array}$$

where $G_{1}^{-1}$ is the quantile function associated with the distribution of Z|A=1 , $G_{0}$ is the cumulative distribution function for Z|A=0 and $F_{0}^{-1}$ the quantile function of Y|A=0 .

**Figure 5.**
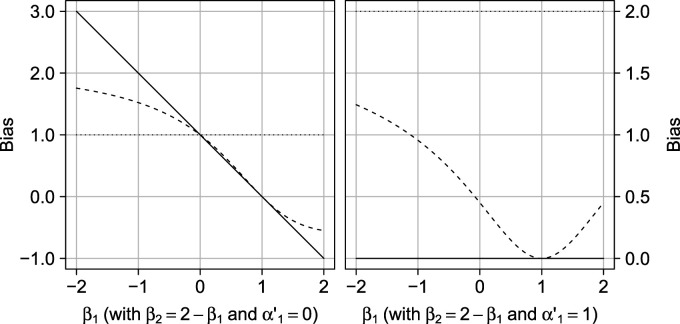
Illustrating of the potential impact of violating additive or quantile–quantile equi-confounding on the bias of the (generalized) difference-in-difference approach. Solid lines represent the difference-in-difference approach; dashed lines the generalized difference-in-difference; dotted lines the bias of a crude analysis, E[Y|A=1]−E[Y|A=0] .

Figure 5 shows, for various parameter specifications, the bias of the (generalized) DiD for the ATT θ . Specifically, $\beta_{1}$ was varied over (−2,2) and $\alpha_{1}^{\prime }$ over {0,1} , while $\beta_{2}$ was set to $2-\beta_{1}$ , and $p_{A}=0.5$ , $\alpha_{0},\alpha_{0}^{\prime },\beta_{0},\theta =0$ and $\alpha_{1},\sigma_{1}^{2},\sigma_{2}^{2},\sigma_{Y}^{2},\sigma_{Z}^{2}=1$ . The figure illustrates that under additive and quantile–quantile equi-confounding the DiD and generalized DiD, respectively, identify the ATT. It also shows that both approaches are sensitive – albeit differently – to violations of their respective assumptions. Interestingly, even in the absence of additive equi-confounding the generalized DiD could be subject to considerable bias (Figure 5, right panel, where the bias for the DiD is $(1-\beta_{1})\alpha_{1}+(1-\beta_{2})\alpha_{2}^{\prime }=2-(\beta_{1}+\beta_{2})=0$ ). Beside the interpretability of its assumptions, an appealing property of the standard DiD approach is that the effects of common causes need not be the same for the NCO and the primary outcome of interest; if the net additive confounding is (close to) the same for the NCO and primary outcome, then the ATT may be (nearly) identified.

### 3.3. Double-negative control approach

#### 3.3.1. Identification

Recent developments in the use of negative controls to adjust for unmeasured confounding leverage multiple negative control variables or proxies of unmeasured common causes.^16–18,4,19^ For example, the next result, due to Miao et al.,^17^ gives a set of conditions sufficient to identify the expected marginal counterfactual outcome E[Y(a)] by leveraging a pair of proxy variables B,Z of an unobserved variable U that renders the counterfactual outcomes independent of the exposure of interest (i.e. conditional exchangeability given U ).

Theorem 6 **The confounding bridge approach**

Suppose that for all levels a of A , the following conditions hold:

∙ *Consistency:* Y(a)=Y if a=A .

∙ *Positivity:* 0<Pr(A=a|B)<1 with probability 1.

∙ *Latent ignorability:* Y(a)⊥⊥(A,B)|U and Z⊥⊥(A,B)|U .

∙ *Confounding bridge assumption:* E[Y|A=a,U]=E[h(Z)|A=a,U] with probability 1 for some h .

∙ *Completeness:* for all g , if E[g(Z)|A=a,B]=0 with probability 1, then Pr(g(Z)=0|A=a)=1 .

Let H(a) be the collection of all h that satisfy E[Y−h(Z)|A=a,B]=0 with probability 1. Then, H(a) is non-empty, and for all h∈H(a) , E[Y(a)]=E[h(Z)] .

**Figure 6.**
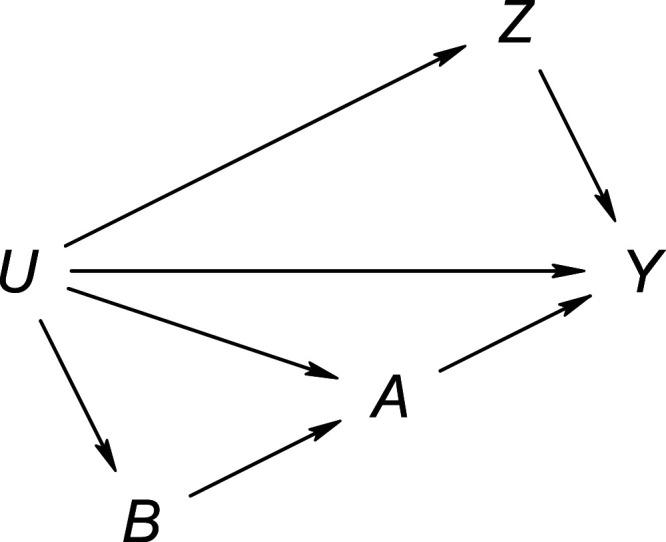
Causal directed acyclic graph with negative control pair satisfying the latent ignorability condition of Theorem 6.

Figure 6 shows a directed acyclic graph that is consistent with the assumptions of Theorem 6. The proxy variables can be seen to be negative control variables in the sense described by Shi et al.,^4^ thus making the confounding bridge approach a (double-)negative control approach. Like the primary exposure-outcome association, the exposure–NCO association is confounded by U . The function h is referred to as a confounding bridge because the confounding bridge assumption indicates that it links the Y - U association with the NCO- U association. The NCE is not part of this link but is meant to help identify it.

The confounding bridge and completeness assumptions can be difficult to grasp. For categorical variables, however, the assumptions are subsumed under the conditions of the next result, due to Miao et al.^16^ and Shi et al.^18^

Theorem 7 **The proximal g-formula for categorical variables**

Let U,B,Z be discrete random variables with finite support such that U has no more categories than B or Z . Suppose that for all levels a of A , the following conditions hold:

∙ *Consistency:* Y(a)=Y if a=A .

∙ *Positivity:* 0<Pr(A=a,B=b) for all categories b of B .

∙ *Latent ignorability:* Y(a)⊥⊥(A,B)|U and Z⊥⊥(A,B)|U .

∙ *Full rank:* Pr(Z|U) and Pr(U|A=a,B) have rank equal to the number of levels of U .

Then, E[Y(a)]=h(Z)Pr(Z) , where $h(Z)=E[Y|A=a,B]\mathrm{Pr}(Z|A=a,B{)}^{-1}$ .

Here, following Miao et al.,^16^ for any categorical variables X,Y,Z , Pr(X|Y,Z) denotes the matrix of probabilities Pr(X=x|Y,Z) with a one-to-one correspondence between rows and categories x of X and a one-to-one correspondence between columns and categories z of Z . Interestingly, the proximal g-formula can also be written as a weighted version of the standard g-formula:

$$\begin{array}{r} E[Y|A=a,B]\mathrm{diag}(W(a))\mathrm{Pr}(B) \end{array}$$

with weights $W(a)=(\mathrm{diag}\mathrm{Pr}(B){)}^{-1}\mathrm{Pr}(Z|A=a,B{)}^{-1}\mathrm{Pr}(Z)$ and diag(W(a)) and diag(B) denoting the diagonal matrices with main diagonals W(a) and B , respectively. In the case that proxy variables B and Z are binary, the expression simplifies to

$$\begin{array}{r} E\{E[WY|A=a,B]\} \end{array}$$

with weights

$$\begin{array}{rl} W & =\frac{\left(1-B\right)}{\mathrm{Pr}(B=0)}\frac{\mathrm{Pr}(Z=1|A,B=1)-\mathrm{Pr}(Z=1)}{\mathrm{Pr}(Z=1|A,B=1)-\mathrm{Pr}(Z=1|A,B=0)}\\ & \;+\frac{-B}{\mathrm{Pr}(B=1)}\frac{\mathrm{Pr}(Z=1|A,B=0)-\mathrm{Pr}(Z=1)}{\mathrm{Pr}(Z=1|A,B=1)-\mathrm{Pr}(Z=1|A,B=0)} \end{array}$$

#### 3.3.2. Sensitivity to assumption violations

Theorem 7 can accommodate any number of categories of U by taking proxy variables with sufficiently many categories, that is, by combining sufficiently many proxies. However, upon increasing the number of proxy variables, the latent ignorability assumption becomes more difficult to satisfy in the sense that Y(a) must be independent of increasingly many proxies given A and U . In this subsection, we consider the sensitivity of the proximal g-formula for violations of latent ignorability as well as of the assumption that U has no more categories than the proxy variables.

In particular, we consider the case where the variables A,Y of interest and the proxy variables B,Z are binary, where U is a pair $\left(U_{1},U_{2}\right)$ of independent binary variables, and where the following models hold:

$$\begin{array}{r} \begin{array}{rl} U_{1} & ∼\mathrm{Bernoulli}(1/2)\\ U_{2}|U_{1} & ∼\mathrm{Bernoulli}(\rho )\\ B|U_{1},U_{2} & ∼\mathrm{Bernoulli}(\mathrm{expit}\{\alpha_{0}+U_{1}+U_{2}\})\\ A|U_{1},U_{2},B & ∼\mathrm{Bernoulli}(\mathrm{expit}\{\beta_{0}+U_{1}+\beta_{1}U_{2}+B\})\\ Z|U_{1},U_{2},B,A & ∼\mathrm{Bernoulli}(\mathrm{expit}\{\gamma_{0}+U_{1}-1/2U_{2}+\gamma_{1}A\})\\ Y|U_{1},U_{2},B,A,Z & ∼\mathrm{Bernoulli}(\mathrm{expit}\{\theta_{0}+U_{1}+U_{2}+Z+\theta_{1}B\}) \end{array} \end{array}$$

where expit(x)=1/(1+exp[−x]) for all x . Intercepts $\alpha_{0},\beta_{0},\gamma_{0},\theta_{0}$ were chosen to ensure that Pr(B=1)=Pr(A=1)=1/2 and Pr(Z=1)=Pr(Y=1)=1/5 . We let $\rho =0,\beta_{1}=1,\gamma_{1}=0,\theta_{1}=0$ by default. In scenario A, instead of taking $\beta_{1}=1,\rho =0$ , we vary $\beta_{1}$ over (−4,4) under ρ=1/2 to violate the full rank assumption, which implies that U has no more categories than B or Z . In scenario B, instead of taking $\gamma_{1}=0$ , we violate the latent ignorability assumption by varying $\gamma_{1}$ over (−4,4) (i.e. Z is not a negative control outcome). In scenario C, we violate the same assumption, now by varying $\theta_{1}$ over (−4,4) (i.e. B is not a negative control exposure) instead of taking $\theta_{1}=0$ .

**Figure 7.**
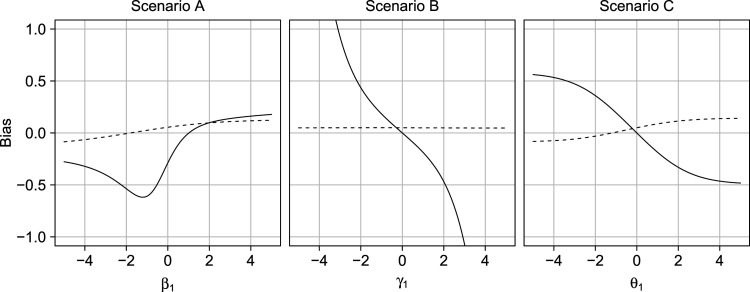
Bias of crude approach (dashed) and proximal g-formula (solid) under violations of the cardinality assumption (scenario A), negative control outcome condition (scenario B), or negative control exposure condition (scenario C).

Figure 7 gives the bias of the proximal g-formula for the ATE E[Y(1)−Y(0)] for all scenarios. Also shown are the differences between the crude risk differences E[Y|A=1]−E[Y|A=0] and the ATE. The bias is zero under the default parameters, which are consistent with the assumptions of Theorem 7. The figure also illustrates that violations of these unverifiable assumptions can have a large impact on the validity of the double-negative control approach.

In an other study, Vlassis et al.^20^ found the bias of the crude risk difference to be consistently smaller than that of the proximal g-formula. Our results demonstrate that in some settings, the proximal g-formula results in considerably more bias than what would result from ignoring unmeasured confounding.

## 4. Conclusion

Negative controls have gained increasing interest in addressing concerns about the validity of a study. The literature on the topic has tended to consider increasingly ambitious tasks, from confounding detection to full identification of causal effects, typically at the cost of stronger and arguably more complex assumptions. Efforts have been made to introduce negative controls to a broader audience and ensure they are adopted in epidemiological practice.^4^ However, little attention has yet been given to the methods’ assumptions and the potential impact of assumption violations. While the assumptions may be tenable enough in some specific cases to justify an application, in other situations substantial violations are possible. We have illustrated that assumption violations, some of which are likely even in very simple settings, may have a considerable impact on the validity of the negative control approach, thereby limiting its utility.

We stress the other methods commonly used to analyse observational data (e.g. covariate adjustment through regression analysis or instrumental variable methods) may also be sensitive to violations of their assumptions. However, a comparison between these methods and methods using negative controls is beyond the scope of this work. Researchers should decide on a case-by-case base of which methods the assumptions appear most plausible and thus which method appears most appropriate. Another aspect that should be considered on a case-by-case base is the magnitude that could arise due to violations of the assumptions underlying negative control methods. The illustrations presented here are based on arbitrary parameter values chosen such that they illustrate the relative bias contributions. However, we do not claim these are necessarily appropriate for a particular study. Considerations about the appropriateness and possible violation of the assumptions of negative control methods are, to a large extent, context-dependent.

Despite the possible abundance of negative controls, their routine use in epidemiological practice may fail to strengthen evidence about exposure–outcome effects unless it can be safely assumed that assumption violations are absent or else if the robustness against these violations is well understood. Given the potential impact of assumption violations, it may sometimes be desirable to replace strong conditions for identification with weaker conditions that are easier to verify, even when these weaker conditions imply at most partial identification. Future research in this area may broaden the applicability of negative controls and in turn make them more suited for routine use in epidemiological practice. When they are used, we advise that researches consider the results of their applications carefully and explicitly in light of the methods’ limitations and assumptions.

## Supplemental Material

sj-pdf-1-smm-10.1177_09622802231181230 - Supplemental material for Negative controls: Concepts and caveats Click here for additional data file. Supplemental material, sj-pdf-1-smm-10.1177_09622802231181230 for Negative controls: Concepts and caveats by Bas BL Penning de Vries and Rolf HH Groenwold in Statistical Methods in Medical Research
