# Supplementary material for: Negative controls: Concepts and caveats
Source: Stat Methods Med Res. 2023 Jun 20;32(8):1576–87. doi: 10.1177/09622802231181230 (PMC10515451; doi:10.1177/09622802231181230)
Supplement: sj-pdf-1-smm-10.1177_09622802231181230 - Supplemental material for Negative controls: Concepts and caveats [file sj-pdf-1-smm-10.1177_09622802231181230.pdf]

Supplementary material to 'Negative controls: concepts and caveats'

Supplementary Appendix A: Identifiability of the direction of bias using an NCO/NCE

Theorem (Identification of the direction of bias using an NCO/NCE). *Suppose the following conditions hold:*

- Latent ignorability for some scalar U: $Z \perp\!\!\!\perp (A, Y)|U$ *and* $Y \perp\!\!\!\perp A|U$.
- Primary exposure model: $A = \alpha_0 + \alpha_1 U + \epsilon$, $\epsilon \perp\!\!\!\perp U$, $\mathbb{E}[\epsilon] = 0$.
- Primary outcome model: $Y = \gamma_0 + \gamma_1 U + \theta A + \varepsilon$, $\varepsilon \perp\!\!\!\perp (A, U)$, $\mathbb{E}[\varepsilon] = 0$.
- NCO/NCE model: $Z = \beta_0^* + \beta_1^* U + \delta$, $\delta \perp\!\!\!\perp (A, Y, U), \mathbb{E}[\delta] = 0$.

Then, $\hat{\theta} - \theta$ has the same sign as

$$\frac{\text{Cov}(Y, Z)}{\text{Cov}(A, Z)} - \hat{\theta}.$$

Proof. For the ordinary least squares coefficient $\hat{\theta} = \text{Cov}(Y, A)/\text{Var}(A)$ in the regression of Y on A, we have

$$\begin{aligned} \hat{\theta} - \theta &= \gamma_1 \frac{\text{Cov}(U, A)}{\text{Var}(A)} && \text{(by the primary outcome model)} \\ &= \gamma_1 \alpha_1 \frac{\text{Var}(U)}{\text{Var}(A)}. && \text{(by the primary exposure model)} \end{aligned}$$

Note that $\text{Var}(\mathbb{E}[A|U]) = \alpha_1^2 \text{Var}(U)$ and, by the law of total variance, $\text{Var}(A) = \text{Var}(\mathbb{E}[A|U]) + \mathbb{E}[\text{Var}(A|U)]$. Thus, $\text{Var}(A) - \mathbb{E}[\text{Var}(A|U)] = \alpha_1^2 \text{Var}(U)$

$$\hat{\theta} - \theta = \frac{\gamma_1}{\alpha_1} \frac{\text{Var}(A) - \mathbb{E}[\text{Var}(A|U)]}{\text{Var}(A)}.$$

The fraction $(\text{Var}(A) - \mathbb{E}[\text{Var}(A|U)])/\text{Var}(A)$ can be interpreted as the proportion of variance of A that is explained by U. By the law of total variance, the fraction is bounded by 0 and 1.

Next, note that

$$\begin{aligned} \frac{\text{Cov}(Y, Z)}{\text{Cov}(A, Z)} &= \frac{\gamma_1 \text{Cov}(Z, U) + \theta \text{Cov}(A, Z)}{\text{Cov}(A, Z)} && \text{(by the primary outcome model)} \\ &= \gamma_1 \frac{\text{Cov}(Z, U)}{\text{Cov}(A, Z)} + \theta \\ &= \gamma_1 \frac{\text{Cov}(Z, U)}{\alpha_1 \text{Cov}(U, Z)} + \theta && \text{(by the primary exposure model)} \\ &= \frac{\gamma_1}{\alpha_1} + \theta. \end{aligned}$$

Hence,

$$\hat{\theta} - \theta = \left(\frac{\text{Cov}(Y, Z)}{\text{Cov}(A, Z)} - \theta \right) \frac{\text{Var}(A) - \mathbb{E}[\text{Var}(A|U)]}{\text{Var}(A)},$$

$$\theta = \frac{\hat{\theta} - \lambda \frac{\text{Cov}(Y, Z)}{\text{Cov}(A, Z)}}{1 - \lambda}$$

$$\hat{\theta} - \theta = \left(\frac{\mathrm{Cov}(Y, Z)}{\mathrm{Cov}(A, Z)} - \hat{\theta} \right) \frac{\lambda}{1 - \lambda}$$

where $\lambda = (\mathrm{Var}(A) - \mathbb{E}[\mathrm{Var}(A|U)])/\mathrm{Var}(A)$. Clearly, since $\lambda \in [0, 1]$ the sign of the bias $\hat{\theta} - \theta$ is identified by $\mathrm{Cov}(Y, Z)/\mathrm{Cov}(A, Z) - \hat{\theta}$. □

Remark (No identifiability of the direction of bias when Z is not an NCE)**.** *Consider the following models*

$$\begin{aligned}
&U \sim \mathrm{Normal}(\mathbb{E}[U], \mathrm{Var}(U)),\\
&A = \alpha_0 + \alpha_1 U + \epsilon,\ \epsilon|U \sim \mathrm{Normal}(0, \mathrm{Var}(\epsilon)),\\
&Z = \beta_0^* + \beta_1^* U + \delta,\ \delta|(U, A) \sim \mathrm{Normal}(0, \mathrm{Var}(\delta)),\\
&Y = \gamma_0 + \gamma_1 U + \gamma_2 Z + \theta A + \varepsilon,\ \varepsilon|(Z, A, U) \sim \mathrm{Normal}(0, \mathrm{Var}(\varepsilon)),
\end{aligned}$$

which are compatible with those of the above theorem if $\gamma_2 = 0$. If $\gamma_2 \neq 0$, then the Latent ignorability condition is violated because $Z \not\perp\!\!\!\perp (A, Y)|U$. If it were possible to infer from the distribution of the observables the direction of bias $\hat{\theta} - \theta$, then there exists some function g of the joint distribution F of (A, Y, Z) such that $g(F)[\hat{\theta} - \theta] > 0$. To prove that this is false, it suffices to show that for some F, the bias $\hat{\theta} - \theta$ may be positive and negative, depending on unobservables, so that for all g, we have $g(F)[\hat{\theta} - \theta] \not> 0$.

Consider the models of the previous section with parameters set to the following values to yield multivariate normal distributions G, H:

	G	H		G	H
$\mathrm{Var}(U)$	1	1	α_1	1	1
$\mathbb{E}[U]$	0	0	β_1^*	1	1
$\mathrm{Var}(\epsilon)$	1	1	γ_1	−5	1
α_0	0	0	θ	1	−1
$\mathrm{Var}(\delta)$	1	1	γ_2	3	1
β_0^*	0	0			
$\mathrm{Var}(\varepsilon)$	1	9			
γ_0	0	0			

Given zero means of A, Y, Z, the corresponding covariance matrices

$$\mathrm{Cov}(G) = \begin{bmatrix} 1 & 1 & 1 & -1 \\ 1 & 2 & 1 & 0 \\ 1 & 1 & 2 & 2 \\ -1 & 0 & 2 & 12 \end{bmatrix}, \qquad \mathrm{Cov}(H) = \begin{bmatrix} 1 & 1 & 1 & 1 \\ 1 & 2 & 1 & 0 \\ 1 & 1 & 2 & 2 \\ 1 & 0 & 2 & 12 \end{bmatrix}$$

imply the same distribution for (A, Y, Z), despite the fact that the true effects θ have opposite signs. This shows that in general the direction of bias cannot be identified.

Supplementary Appendix B: Proofs to theorems in section 3

Proof to Theorem 1. For all a,

$$\begin{aligned}
g^{-1}(\beta_0 + \beta_2 y) &= \mathbb{E}[Z|A = 0, Y = y] && \text{(by NCO model)}\\
&= \mathbb{E}[Z|A = 0, Y(0) = y] && \text{(by consistency)}\\
&= \mathbb{E}[Z|A = a, Y(0) = y] && \text{(by exposure-NCO independence given counterfactual outcome)}\\
&= \mathbb{E}[Z|A = a, Y(0) + \theta A - \theta A = y] &&\\
&= \mathbb{E}[Z|A = a, Y(a) = y + \theta a] && \text{(by rank preservation)}
\end{aligned}$$

$$
\begin{aligned}
&= \mathbb{E}[Z|A=a, Y=y+\theta a] && \text{(by consistency)}\\
&= g^{-1}(\beta_0 + \beta_1 a + \beta_2(y+\theta a))\\
&= g^{-1}(\beta_0 + (\beta_1 + \beta_2\theta)a + \beta_2 y),
\end{aligned}
$$

so that, for $a = 1$,

$$
\begin{aligned}
\beta_0 + \beta_2 y &= \beta_0 + (\beta_1 + \beta_2\theta) + \beta_2 y,\\
\theta &= -\beta_1/\beta_2.
\end{aligned}
$$

□

Proof to Theorem 2.

$$
\begin{aligned}
Z &= \beta_0 + \beta_1 Y(0) + \rho(A - \mathbb{E}[A|Y(0)]) + \chi && \text{(by linear NCO model)}\\
&= \beta_0 + \beta_1 Y(0) + \rho(A - \alpha_0 - \alpha_1 Y(0)) + \chi && \text{(by linear exposure model)}\\
&= \beta_0 + \beta_1(Y(A) - \theta A) + \rho(A - \alpha_0 - \alpha_1 Y(A) + \alpha_1\theta A) + \chi && \text{(by rank preservation)}\\
&= (\beta_0 - \rho\alpha_0) + (\rho + [\rho\alpha_1 - \beta_1]\theta)A + (\beta_1 - \rho\alpha_1)Y + \chi, && \text{(by consistency)}\\
\text{and } \mathbb{E}[Z|A,Y] &= (\beta_0 - \rho\alpha_0 + \mathbb{E}[\chi]) + (\rho + [\rho\alpha_1 - \beta_1]\theta)A + (\beta_1 - \rho\alpha_1)Y, && \text{(by linear NCO model)}
\end{aligned}
$$

so that

$$
\beta_1^* = \rho + (\rho\alpha_1 - \beta_1)\theta \text{ and } \beta_2^* = \beta_1 - \rho\alpha_1,
$$

and, in turn, $\theta = (\beta_1^* - \rho)/\beta_2^*$. □

Proof to Theorem 3. We have

$$
\begin{aligned}
\mathbb{E}[Z|A=1-a] &= \mathbb{E}\{\mathbb{E}[Z|A=1-a, Y(a)]|A=1-a\}\\
&= \mathbb{E}\{\mathbb{E}[Z|A=a, Y(a)]|A=1-a\}\\
& \qquad \text{(by exposure-NCO independence given counterfactual outcome)}\\
&= \mathbb{E}[Z|A=a, Y=0]\Pr(Y(a)=0|A=1-a) +\\
&\qquad + \mathbb{E}[Z|A=a, Y=1]\Pr(Y(a)=1|A=1-a) && \text{(by consistency)}\\
&= \mathbb{E}[Z|A=a, Y=0] + \{\mathbb{E}[Z|A=a, Y=1] - \mathbb{E}[Z|A=a, Y=0]\}\Pr(Y(a)=1|A=1-a),
\end{aligned}
$$

so that

$$
\Pr(Y(a)=1|A=1-a) = \frac{\mathbb{E}[Z|A=1-a] - \mathbb{E}[Z|A=a, Y=0]}{\mathbb{E}[Z|A=a, Y=1] - \mathbb{E}[Z|A=a, Y=0]}.
$$

It follows that

$$
\begin{aligned}
\mathbb{E}[Y(a)] &= \mathbb{E}[Y(a)|A=a]\Pr(A=a) + \mathbb{E}[Y(a)|A=1-a]\Pr(A=1-a)\\
&= \mathbb{E}[Y|A=a]\Pr(A=a) + \mathbb{E}[Y(a)|A=1-a]\Pr(A=1-a) && \text{(by consistency)}\\
&= \mathbb{E}[Y|A=a]\Pr(A=a) + \frac{\mathbb{E}[Z|A=1-a] - \mathbb{E}[Z|A=a, Y=0]}{\mathbb{E}[Z|A=a, Y=1] - \mathbb{E}[Z|A=a, Y=0]}\Pr(A=1-a).
\end{aligned}
$$

□

Proof to Theorem 4.

$$
\begin{aligned}
\mathbb{E}[Y(1) - Y(0)|A=1] &= \mathbb{E}[Y(1)|A=1] - \mathbb{E}[Y(0)|A=1]\\
&= \mathbb{E}[Y|A=1] - \mathbb{E}[Y(0)|A=1] && \text{(by consistency)}\\
&= \mathbb{E}[Y|A=1] - (\mathbb{E}[Y(0)|A=1] - \mathbb{E}[Y(0)|A=0]) - \mathbb{E}[Y(0)|A=0]\\
&= \mathbb{E}[Y|A=1] - (\mathbb{E}[N|A=1] - \mathbb{E}[N|A=0]) - \mathbb{E}[Y(0)|A=0]\\
& && \text{(by additive equi-confounding)}\\
&= (\mathbb{E}[Y|A=1] - \mathbb{E}[Y|A=0]) - (\mathbb{E}[N|A=1] - \mathbb{E}[N|A=0]) && \text{(by consistency)}
\end{aligned}
$$

□

Proof to Theorem 5. By quantile-quantile equi-confounding, we have, for all $p \in [0,1]$,

$$\begin{aligned} F_0(F_1^{-1}(p)) &= G_0(G_1^{-1}(p)), \\ F_0^{-1}(F_0(F_1^{-1}(p))) &= F_0^{-1}(G_0(G_1^{-1}(p))), \\ F_1^{-1}(p) &= F_0^{-1}(G_0(G_1^{-1}(p))). && \text{(under strictly monotonic } F_1) \end{aligned}$$

Note that the right-hand side of the above equality is a functional of observables because $F_0(y) = \Pr(Y(0) \le y | A = 0) = \Pr(Y \le y | A = 0)$ by consistency. Now, letting $V \sim \text{Uniform}[0,1]$, we have that $F_1^{-1}(V) \sim Y(0)|A = 1$ by the Probability Integral Transform theorem, and so

$$\mathbb{E}[Y(0)|A = 1] = \mathbb{E}[F_0^{-1}(G_0(G_1^{-1}(V)))].$$

□

Proof to Theorem 6. Let h be the function satisfying $\mathbb{E}[Y|A = a, U] = \mathbb{E}[h(Z)|A = a, U]$ with probability 1 (and note that this function exists by the confounding bridge assumption). Let $\mathcal{U} = \{u : \mathbb{E}[Y|A = a, U = u] = \mathbb{E}[h(Z)|A = a, U = u]\}$, so that $\Pr(U \in \mathcal{U}) = 1$ and

$$\begin{aligned} \mathbb{E}[Y(a)] &= \mathbb{E}[Y(a)|U \in \mathcal{U}] \\ &= \mathbb{E}\{\mathbb{E}[Y(a)|U]|U \in \mathcal{U}\} \\ &= \mathbb{E}\{\mathbb{E}[Y(a)|A = a, U]|U \in \mathcal{U}\} && \text{(since } Y(a) \perp\!\!\!\perp A|U \text{ by latent ignorability)} \\ &= \mathbb{E}\{\mathbb{E}[Y|A = a, U]|U \in \mathcal{U}\} && \text{(by consistency)} \\ &= \mathbb{E}\{\mathbb{E}[h(Z)|A = a, U]|U \in \mathcal{U}\} \\ &= \mathbb{E}\{\mathbb{E}[h(Z)|U]|U \in \mathcal{U}\} && \text{(since } Z \perp\!\!\!\perp A|U \text{ by latent ignorability)} \\ &= \mathbb{E}[h(Z)|U \in \mathcal{U}] \\ &= \mathbb{E}[h(Z)]. \end{aligned}$$

Next, note that by the confounding bridge assumption, for all $U \in \mathcal{U}$,

$$\begin{aligned} \mathbb{E}[Y|A = a, U] &= \mathbb{E}[h(Z)|A = a, U] \\ \mathbb{E}[Y|A = a, B, U] &= \mathbb{E}[h(Z)|A = a, B, U], && \text{(by latent ignorability)} \end{aligned}$$

so that

$$\begin{aligned} \mathbb{E}\{\mathbb{E}[Y|A = a, B, U]|A = a, B\} &= \mathbb{E}\{\mathbb{E}[h(Z)|A = a, B, U]|A = a, B\} \\ \mathbb{E}[Y|A = a, B] &= \mathbb{E}[h(Z)|A = a, B], \\ \mathbb{E}[Y - h(Z)|A = a, B] &= 0. \end{aligned}$$

Let $\mathcal{H}(a)$ be the collection of all h' satisfying $\mathbb{E}[Y - h'(Z)|A = a, B] = 0$ with probability 1. Now, for any $h' \in \mathcal{H}(a)$, we must have

$$\mathbb{E}[h(Z) - h'(Z)|A = a, B] = 0.$$

But from completeness, with $g(Z) = h(Z) - h'(Z)$, it follows that $h(Z) = h'(Z)$ with probability 1. This concludes the proof. □

Proof to Theorem 7. Since $Z \perp\!\!\!\perp (A, B)|U$, we have $\Pr(\boldsymbol{Z}|A = a, \boldsymbol{B}) = \Pr(\boldsymbol{Z}|\boldsymbol{U}) \Pr(\boldsymbol{U}|A = a, \boldsymbol{B})$. Since matrices $\Pr(\boldsymbol{Z}|\boldsymbol{U})$ and $\Pr(\boldsymbol{U}|A = a, \boldsymbol{B})$ are of full rank, $\Pr(\boldsymbol{Z}|A = a, \boldsymbol{B})$ is of full rank and has left or right inverse $\Pr(\boldsymbol{Z}|A = a, \boldsymbol{B})^{-1}$. Let $h(\boldsymbol{Z}) = \mathbb{E}[Y|A = a, \boldsymbol{B}] \Pr(\boldsymbol{Z}|A = a, \boldsymbol{B})^{-1}$ and observe that

$$\begin{aligned} h(\boldsymbol{Z}) &= \mathbb{E}[Y(a)|A = a, \boldsymbol{B}] \Pr(\boldsymbol{Z}|A = a, \boldsymbol{B})^{-1} && \text{(by consistency)} \\ &= \mathbb{E}[Y(a)|\boldsymbol{U}] \Pr(\boldsymbol{U}|A = a, \boldsymbol{B}) \Pr(\boldsymbol{Z}|A = a, \boldsymbol{B})^{-1} && \text{(since } Y(a) \perp\!\!\!\perp (A, B)|U) \\ &= \mathbb{E}[Y(a)|\boldsymbol{U}] \Pr(\boldsymbol{U}|A = a, \boldsymbol{B})[\Pr(\boldsymbol{Z}|\boldsymbol{U}) \Pr(\boldsymbol{U}|A = a, \boldsymbol{B})]^{-1} && \text{(since } Z \perp\!\!\!\perp (A, B)|U) \end{aligned}$$

$$= \mathbb{E}[Y(a)|\boldsymbol{U}] \Pr(\boldsymbol{U}|A = a, \boldsymbol{B}) \Pr(\boldsymbol{U}|A = a, \boldsymbol{B})^{-1} \Pr(\boldsymbol{Z}|\boldsymbol{U})^{-1}$$
$$= \mathbb{E}[Y(a)|\boldsymbol{U}] \Pr(\boldsymbol{Z}|\boldsymbol{U})^{-1}.$$

It follows that $\mathbb{E}[Y(a)|\boldsymbol{U}] = h(\boldsymbol{Z}) \Pr(\boldsymbol{Z}|\boldsymbol{U})$ and in turn $\mathbb{E}[Y(a)] = \mathbb{E}[Y(a)|\boldsymbol{U}] \Pr(\boldsymbol{U}) = h(\boldsymbol{Z}) \Pr(\boldsymbol{Z})$, as desired.

□

Supplementary Appendix C: Derivation of expressions in section 3.1

C.1 Implications of models (1)

Expression of the COCA

An implementation of the COCA by ordinary least squares under the linear NCO model $\mathbb{E}[Z|A, Y] = \beta_0 + \beta_1 A + \beta_2 Y$, identifies the following quantity

$$\hat{\theta} = -\frac{\hat{\beta}_1}{\hat{\beta}_2}$$
$$= -\frac{\text{Cov}(A, Z)\text{Var}(Y) - \text{Cov}(Y, Z)\text{Cov}(A, Y)}{\text{Cov}(Y, Z)\text{Var}(A) - \text{Cov}(A, Z)\text{Cov}(A, Y)},$$

where

$$\text{Var}(Y) = \text{Var}(A)\alpha_1^2 + \sigma_Y^2 + \sigma_\theta^2\text{Var}(A) + \sigma_\theta^2\mathbb{E}[A]^2 + \mathbb{E}[\theta]^2\text{Var}(A) + 2\text{Var}(A)\alpha_1\mathbb{E}[\theta],$$
$$\text{Var}(Z) = \text{Var}(A)\alpha_1^2\gamma_1^2 + \gamma_1^2\sigma_Y^2 + \sigma_Z^2,$$
$$\text{Cov}(A, Y) = \text{Var}(A)\alpha_1 + \text{Var}(A)\mathbb{E}[\theta],$$
$$\text{Cov}(A, Z) = \text{Var}(A)\alpha_1\gamma_1,$$
$$\text{Cov}(Y, Z) = \gamma_1(\text{Var}(A)\alpha_1^2 + \sigma_Y^2 + \text{Var}(A)\alpha_1\mathbb{E}[\theta]).$$

Deterministic relation between $\mathbb{E}[\theta]$ and $\text{Var}[\theta]$ given observed data distribution

From the expressions of the variances and covariates above, for arbitrary $\mathbb{E}[\theta], \text{Var}(A)$, it follows that

$$\alpha_1 = \frac{\text{Cov}(A, Y) - \text{Var}(A)\mathbb{E}[\theta]}{\text{Var}(A)},$$
$$\alpha_0 = \mathbb{E}[Y] - (\alpha_1 + \mathbb{E}[\theta])\mathbb{E}[A],$$
$$\gamma_1 = \frac{\text{Cov}(A, Z)}{\text{Var}(A)\alpha_1},$$
$$\gamma_0 = \mathbb{E}[Z] - (\alpha_0\gamma_1 + \alpha_1\gamma_1\mathbb{E}[A]),$$
$$\sigma_Y^2 = \frac{\text{Cov}(Y, Z) - \gamma_1(\text{Var}(A)\alpha_1^2 + \text{Var}(A)\alpha_1\mathbb{E}[\theta])}{\gamma_1},$$
$$\sigma_\theta^2 = \frac{\text{Var}(Y) - [\text{Var}(A)\alpha_1^2 + \sigma_Y^2 + \text{Var}(A)\mathbb{E}[\theta]^2 + 2\text{Var}(A)\alpha_1\mathbb{E}[\theta]]}{\text{Var}(A) + \mathbb{E}[A]^2},$$
$$\sigma_Z^2 = \text{Var}(Z) - (\text{Var}(A)\alpha_1^2\gamma_1^2 + \gamma_1^2\sigma_Y^2),$$

provided that $\text{Var}(A), \alpha_1, \gamma_1 \neq 0$, and $\sigma_Y^2, \sigma_\theta^2, \sigma_Z^2 \geq 0$. Note that the right-hand sides of every equality are expressed only in terms of functionals of the available data distribution and the left-hand sides of the equalities above it. It follows that we have a deterministic relationship between $\text{Var}(\theta)$ and $\mathbb{E}[\theta]$ given the observed data distribution of (A, Y, Z). In fact, the relationship is linear:

$$\sigma_\theta^2 = \frac{\text{Var}(Y)\text{Cov}(A, Z) - \text{Cov}(A, Y)\text{Cov}(Y, Z)}{(\text{Var}(A) + \mathbb{E}[A]^2)\text{Cov}(A, Z)} - \frac{\text{Cov}(A, Y)\text{Cov}(A, Z) - \text{Var}(A)\text{Cov}(Y, Z)}{(\text{Var}(A) + \mathbb{E}[A]^2)\text{Cov}(A, Z)}\mathbb{E}[\theta].$$
$$= \frac{\text{Var}(A)\text{Var}(Y) - \text{Cov}(A, Y)^2}{(\text{Var}(A) + \mathbb{E}[A]^2)\text{Cov}(A, Z)}(\hat{\beta}_1 - \hat{\beta}_2\mathbb{E}[\theta]).$$

The distribution of $Z|A, Y$

First note that

$$\begin{aligned}
\mathbb{E}[\theta|Y, A=0] &= \mathbb{E}[\theta|Y(0), A=0] && \text{(by consistency)} \\
&= \mathbb{E}[\theta|A=0] && \text{(since } Y(0) \perp\!\!\!\perp \theta|A) \\
&= \mathbb{E}[\theta]. && \text{(since } \theta \perp\!\!\!\perp A)
\end{aligned}$$

Next, for arbitrary a, consider $\mathbb{E}[\theta|Y, A=a]$ and note that $(\theta, Y)|A=a$ takes a bivariate normal distribution with means

$$\begin{aligned}
\mathbb{E}[\theta|A=a] &= \mathbb{E}[\theta], \\
\mathbb{E}[Y|A=a] &= \mathbb{E}[Y(A)|A=a] && \text{(by consistency)} \\
&= \mathbb{E}[Y(0)+\theta A|A=a] \\
&= \alpha_0 + \alpha_1 a + \mathbb{E}[\theta],
\end{aligned}$$

variances

$$\begin{aligned}
\text{Var}(\theta|A=a) &= \sigma_\theta^2, && \text{(since } \theta \perp\!\!\!\perp A) \\
\text{Var}(Y|A=a) &= \text{Var}(Y(A)|A=a) && \text{(by consistency)} \\
&= \text{Var}(Y(0)+\theta A|A=a) \\
&= \text{Var}(Y(0)|A=a) + \text{Var}(\theta) && \text{(since } Y(0) \perp\!\!\!\perp \theta|A \text{ and } \theta \perp\!\!\!\perp A) \\
&= \sigma_Y^2 + \sigma_\theta^2,
\end{aligned}$$

and correlation

$$\begin{aligned}
\text{Cor}(Y, \theta|A=a) &= \sqrt{\frac{\text{Cov}^2(Y, \theta|A=a)}{\text{Var}(Y|A=a)\text{Var}(\theta|A=a)}} \\
&= \sqrt{\frac{\mathbb{E}[(Y-\mathbb{E}[Y|A=a])(\theta-\mathbb{E}[\theta|A=a])|A=a]^2}{\text{Var}(Y|A=a)\text{Var}(\theta|A=a)}} \\
&= \sqrt{\frac{\mathbb{E}[(Y(A)-\mathbb{E}[Y(A)|A=a])(\theta-\mathbb{E}[\theta|A=a])|A=a]^2}{\text{Var}(Y|A=a)\text{Var}(\theta|A=a)}} && \text{(by consistency)} \\
&= \sqrt{\frac{\mathbb{E}[(Y(0)+\theta A-\mathbb{E}[Y(0)+\theta A|A=a])(\theta-\mathbb{E}[\theta|A=a])|A=a]^2}{\text{Var}(Y|A=a)\text{Var}(\theta|A=a)}} \\
&= \sqrt{\frac{\mathbb{E}[((Y(0)-\mathbb{E}[Y(0)|A=a])+(\theta A-\mathbb{E}[\theta|A=a]))(\theta-\mathbb{E}[\theta|A=a])|A=a]^2}{\text{Var}(Y|A=a)\text{Var}(\theta|A=a)}} \\
&= \sqrt{\frac{[\text{Cov}(Y(0), \theta|A=a)+\text{Cov}(\theta A, \theta|A=a)]^2}{\text{Var}(Y|A=a)\text{Var}(\theta|A=a)}} \\
&= \sqrt{\frac{a^2\text{Var}(\theta|A=a)^2}{\text{Var}(Y|A=a)\text{Var}(\theta|A=a)}} && \text{(since } Y(0) \perp\!\!\!\perp \theta|A) \\
&= a\sqrt{\frac{\sigma_\theta^2}{\sigma_Y^2+\sigma_\theta^2}}.
\end{aligned}$$

Therefore,

$$\mathbb{E}[\theta|Y, A=a] = \mathbb{E}[\theta] + \sqrt{\frac{\sigma_\theta^2}{\sigma_Y^2+\sigma_\theta^2}}\frac{\sigma_\theta^2}{\sigma_Y^2}[-(\alpha_0+\mathbb{E}[\theta])a - \alpha_1 a^2 + aY]$$

(DeGroot and Schervisch, 2012, Theorem 5.10.4, p. 340).

Hence,

$$\mathbb{E}[\theta|Y, A] = \mathbb{E}[\theta] + \sqrt{\frac{\sigma_\theta^2}{\sigma_Y^2+\sigma_\theta^2}}\frac{\sigma_\theta^2}{\sigma_Y^2}[-(\alpha_0+\mathbb{E}[\theta])A - \alpha_1 A^2 + AY]$$

and

$$
\begin{aligned}
Z &= \gamma_0 + \gamma_1 Y(0) + \varepsilon, \;\; \varepsilon|(A, \theta, Y(0)) \sim \text{Normal}(0, \sigma_Z^2) \\
&= \gamma_0 + \gamma_1 (Y(A) - \theta A) + \varepsilon \\
&= \gamma_0 + \gamma_1 (Y - \theta A) + \varepsilon && \text{(by consistency)} \\
&= \gamma_0 - \gamma_1 \theta A + \gamma_1 Y + \varepsilon,
\end{aligned}
$$

so that $Z|A, Y$ has a normal distribution with mean

$$
\begin{aligned}
\mathbb{E}[Z|A, Y] &= \gamma_0 - \gamma_1 \mathbb{E}[\theta|Y, A] A + \gamma_1 Y \\
&= \gamma_0 - \gamma_1 A \left[\mathbb{E}[\theta] + \sqrt{\frac{\sigma_\theta^2}{\sigma_Y^2 + \sigma_\theta^2}} \frac{\sigma_\theta^2}{\sigma_Y^2} [-(\alpha_0 + \mathbb{E}[\theta])A - \alpha_1 A^2 + AY] \right] + \gamma_1 Y \\
&= \gamma_0 - \gamma_1 \mathbb{E}[\theta] A + \sqrt{\frac{\sigma_\theta^2}{\sigma_Y^2 + \sigma_\theta^2}} \frac{\sigma_\theta^2}{\sigma_Y^2} [(\alpha_0 + \mathbb{E}[\theta])\gamma_1 A^2 + \alpha_1 \gamma_1 A^3 - \gamma_1 A^2 Y] + \gamma_1 Y \\
&= \beta_0^* + \beta_1^* A + \beta_2^* A^2 + \beta_3^* A^3 + \beta_4^* Y + \beta_5^* A^2 Y,
\end{aligned}
$$

where

$$
\begin{aligned}
\beta_0^* &= \gamma_0, \\
\beta_1^* &= -\gamma_1 \mathbb{E}[\theta], \\
\beta_2^* &= \sqrt{\frac{\sigma_\theta^2}{\sigma_Y^2 + \sigma_\theta^2}} \frac{\sigma_\theta^2}{\sigma_Y^2} (\alpha_0 + \mathbb{E}[\theta]) \gamma_1, \\
\beta_3^* &= \sqrt{\frac{\sigma_\theta^2}{\sigma_Y^2 + \sigma_\theta^2}} \frac{\sigma_\theta^2}{\sigma_Y^2} \alpha_1 \gamma_1, \\
\beta_4^* &= \gamma_1, \\
\beta_5^* &= -\sqrt{\frac{\sigma_\theta^2}{\sigma_Y^2 + \sigma_\theta^2}} \frac{\sigma_\theta^2}{\sigma_Y^2} \gamma_1,
\end{aligned}
$$

so $\mathbb{E}[\theta] = -\beta_1^*/\beta_4^*$ if $\beta_4^* \neq 0$. Therefore, with a continuous primary outcome and non-binary exposure, the rank preservation assumption can sometimes be dropped whilst maintaining identifiability. If A is binary, we have $\mathbb{E}[Z|A, Y] = \beta_0^* + (\beta_1^* + \beta_2^* + \beta_3^*)A + \beta_4^* Y + \beta_5^* AY$, where

$$
\begin{aligned}
(\beta_1^* + \beta_2^* + \beta_3^*) &= \gamma_1 \sqrt{\frac{\sigma_\theta^2}{\sigma_Y^2 + \sigma_\theta^2}} \frac{\sigma_\theta^2}{\sigma_Y^2} (\alpha_0 + \alpha_1) + \gamma_1 \left(\sqrt{\frac{\sigma_\theta^2}{\sigma_Y^2 + \sigma_\theta^2}} \frac{\sigma_\theta^2}{\sigma_Y^2} - 1 \right) \mathbb{E}[\theta] \\
&= -\beta_5^* (\alpha_0 + \alpha_1) - (\beta_4^* + \beta_5^*) \mathbb{E}[\theta].
\end{aligned}
$$

This suggests a test for violations of rank preservation since the interaction term coefficient β_5^* is zero if and only if $\text{Var}(\theta) = 0$ or $\beta_4^* = 0$. Provided that $\beta_4^* \neq 0$, a valid test of the null hypothesis $\beta_5^* = 0$ is thus a valid test of rank preservation under the above models.

C.2 Implications of models (3)

Under models 3, we have the following variances and covariances:

$$
\begin{aligned}
\text{Var}(A) &= \alpha_1^2 \text{Var}(U_1) + \alpha_2^2 \text{Var}(U_2) + \sigma_A^2, \\
\text{Var}(Y) &= (1 + \theta\alpha_1)^2 \text{Var}(U_1) + (1 + \theta\alpha_2)^2 \text{Var}(U_2) + \theta^2 \sigma_A^2, \\
\text{Var}(Z) &= (\alpha_1')^2 \text{Var}(U_1) + (\alpha_2')^2 \text{Var}(U_1) + \sigma_Z^2, \\
\text{Cov}(A, Y) &= (1 + \theta\alpha_1)\alpha_1 \text{Var}(U_1) + (1 + \theta\alpha_2)\alpha_2 \text{Var}(U_2) + \theta \sigma_A^2, \\
\text{Cov}(A, Z) &= \alpha_1 \alpha_1' \text{Var}(U_1) + \alpha_2 \alpha_2' \text{Var}(U_2), \\
\text{Cov}(Y, Z) &= (1 + \theta\alpha_1)\alpha_1' \text{Var}(U_1) + (1 + \theta\alpha_2)\alpha_2' \text{Var}(U_2)
\end{aligned}
$$

and means

$$\begin{aligned}\mathbb{E}[A] &= \alpha_0 + \alpha_1\mathbb{E}[U_1] + \alpha_2\mathbb{E}[U_2],\\ \mathbb{E}[Y] &= \theta\alpha_0 + (1+\theta\alpha_1)\mathbb{E}[U_1] + (1+\theta\alpha_2)\mathbb{E}[U_2],\\ \mathbb{E}[Z] &= \alpha_0 + \alpha_1\mathbb{E}[U_1] + \alpha_2\mathbb{E}[U_2].\end{aligned}$$

C.3 Partial identification in the presence of classical measurement error in the outcome

Theorem. *Suppose the following conditions hold:*
- Rank preservation: $Y(A) = Y(0) + \theta A$, θ *constant.*
- Exposure-NCO independence given counterfactual outcome: $Z \perp\!\!\!\perp A|Y(0)$.
- NCO model: $Z = \beta_0^* + \beta_1^* Y(0) + \varepsilon$, $\varepsilon \perp\!\!\!\perp (A, Y(0)), \mathbb{E}[\varepsilon] = 0$.
- Classical measurement error: $Y = Y(A) + U$, $U \perp\!\!\!\perp (A, Y(0), Z)$, $\mathbb{E}[U] = 0$.

Then,

$$\theta \in \left[\hat\theta, \hat\theta\left(1 - R^2\frac{1}{1-\mathrm{Cor}^2(A,Y)}\right) + R^2\frac{\mathrm{Var}(Y)}{\mathrm{Cov}(A,Y)}\left(1 - \frac{1}{1-\mathrm{Cor}^2(A,Y)}\right)\right],$$

where $R^2 = 1-\mathbb{E}[\mathrm{Var}(Y|A)]/\mathrm{Var}(Y)$ *is the proportion of variance of* Y *explained by* A*, and* $\hat\theta = -\hat\beta_1/\hat\beta_2$ *and* $\hat\beta_1$ *and* $\hat\beta_1$ *are the ordinary least squares coefficients for* A *and* Y *in a linear regression of* Z *on* A *and* Y*.*

Proof. We have that

$$\begin{aligned}Z &= \beta_0^* + \beta_1^* Y(0) + \varepsilon && \text{(by NCO model)}\\ &= \beta_0^* + \beta_1^*(Y(A) - \theta A) + \varepsilon && \text{(by rank preservation)}\\ &= \beta_0^* + \beta_1^*(Y - U - \theta A) + \varepsilon && \text{(under classical measurement error)}\\ &= \beta_0^* + \beta_1^* Y - \beta_1^* U - \beta_1^*\theta A + \varepsilon,\end{aligned}$$

where $\varepsilon \perp\!\!\!\perp (Y, A, U)$ (since $U \perp\!\!\!\perp \varepsilon|(A, Y(0))$ and $\varepsilon \perp\!\!\!\perp (A, Y(0))$, so that $\varepsilon \perp\!\!\!\perp (Y(0), A, U)$).

Now, let

$$\begin{aligned}\hat\beta_1 &= \frac{\mathrm{Cov}(A,Z)\mathrm{Var}(Y) - \mathrm{Cov}(Y,Z)\mathrm{Cov}(A,Y)}{\mathrm{Var}(A)\mathrm{Var}(Y) - \mathrm{Cov}^2(A,Y)},\\ \hat\beta_2 &= \frac{\mathrm{Cov}(Y,Z)\mathrm{Var}(A) - \mathrm{Cov}(A,Z)\mathrm{Cov}(A,Y)}{\mathrm{Var}(A)\mathrm{Var}(Y) - \mathrm{Cov}^2(A,Y)},\end{aligned}$$

the ordinary least squares coefficients in a linear regression of Z on A and Y. We have

$$\begin{aligned}\mathrm{Cov}(A,Z) &= \beta_1^*(\mathrm{Cov}(A,Y) - \theta\mathrm{Var}(A)),\\ \mathrm{Cov}(Y,Z) &= \beta_1^*(\mathrm{Var}(Y) - \mathrm{Var}(U) - \theta\mathrm{Cov}(A,Y)),\end{aligned}$$

so that

$$\begin{aligned}\hat\beta_1 &= \beta_1^*\left(\frac{\mathrm{Cov}(A,Y)\mathrm{Var}(U)}{\mathrm{Var}(A)\mathrm{Var}(Y) - \mathrm{Cov}^2(A,Y)} - \theta\right),\\ \hat\beta_2 &= \beta_1^*\left(1 - \frac{\mathrm{Var}(A)\mathrm{Var}(U)}{\mathrm{Var}(A)\mathrm{Var}(Y) - \mathrm{Cov}^2(A,Y)}\right)\end{aligned}$$

and in turn

$$\hat\theta = -\frac{\hat\beta_1}{\hat\beta_2}$$

$$= -\frac{\mathrm{Cov}(A,Y)\mathrm{Var}(U) - \theta(\mathrm{Var}(A)\mathrm{Var}(Y) - \mathrm{Cov}^2(A,Y))}{\mathrm{Var}(A)\mathrm{Var}(U) - (\mathrm{Var}(A)\mathrm{Var}(Y) - \mathrm{Cov}^2(A,Y))},$$

$$\theta = \hat{\theta}\left(1 - \frac{\mathrm{Var}(A)\mathrm{Var}(U)}{\mathrm{Var}(A)\mathrm{Var}(Y) - \mathrm{Cov}^2(A,Y)}\right) + \frac{\mathrm{Cov}(A,Y)\mathrm{Var}(U)}{\mathrm{Var}(A)\mathrm{Var}(Y) - \mathrm{Cov}^2(A,Y)}$$

$$= \hat{\theta}\left(1 - \frac{\mathrm{Var}(U)}{\mathrm{Var}(Y)}\frac{1}{1 - \mathrm{Cor}^2(A,Y)}\right) + \frac{\mathrm{Var}(U)}{\mathrm{Var}(Y)}\frac{\mathrm{Var}(Y)}{\mathrm{Cov}(A,Y)}\left(1 - \frac{1}{1 - \mathrm{Cor}^2(A,Y)}\right).$$

By the law of total (conditional) variance,

$$\begin{aligned}
\mathrm{Var}(Y) &= \mathbb{E}[\mathrm{Var}(Y|A)] + \mathrm{Var}(\mathbb{E}[Y|A]) \\
&= \mathbb{E}[\mathrm{Var}(Y|A, Y(0))|A] + \mathbb{E}[\mathrm{Var}(\mathbb{E}[Y|A, Y(0)])|A] + \mathrm{Var}(\mathbb{E}[Y|A]) \\
&= \mathrm{Var}(U) + \mathbb{E}[\mathrm{Var}(\mathbb{E}[Y|A, Y(0)])|A] + \mathrm{Var}(\mathbb{E}[Y|A]).
\end{aligned}$$

Now, define $R^2 = (\mathrm{Var}(Y) - \mathbb{E}[\mathrm{Var}(Y|A)])/\mathrm{Var}(Y)$, the proportion of variance of Y explained by A and observe that

$$R^2 \geq \frac{\mathrm{Var}(U)}{\mathrm{Var}(Y)} \geq 0.$$

Next, define

$$\tilde{\theta}(\lambda) = \hat{\theta}\left(1 - \lambda\frac{1}{1 - \mathrm{Cor}^2(A,Y)}\right) + \lambda\frac{\mathrm{Var}(Y)}{\mathrm{Cov}(A,Y)}\left(1 - \frac{1}{1 - \mathrm{Cor}^2(A,Y)}\right)$$

and note that, because the first derivative of $\tilde{\theta}$ is invariant to changes in λ, $\tilde{\theta}$ is monotonic. Hence

$$\theta \in [\tilde{\theta}(0), \tilde{\theta}(R^2)].$$

□

References

DeGroot, M. and M. Schervish (2012): *Probability and Statistics*, Boston: Pearson, 4th edition.
